# Responsibilities of Medical Colleges

**Published:** 1881-08

**Authors:** W. R. Monroe

**Affiliations:** Baltimore


ARTICLE III.

### RESPONSIBILITIES OF MEDICAL COLLEGES.

BY W. R. MONROE, M. D., BALTIMORE.

The temptation of Medical Institutions to procure large classes of students,and to graduate correspondingly large numbers annually, may arise from several sources : First the motive may be a pecuniary one; to fill the pockets of the professors. To endeavor to make a medical school a money making institution is only to lower the standard of medical instruction in said school. The money loving or money making motive is therefore a base one.

> "O cursed lust of gold ! when for thy sake
> The fool shows up his interest in both worlds,
> First starved in this, then damned in that to come !"

A second motive may arise from a desire for large audiences to inspire the lecturer with eloquence and brilliance, so as to procure eclat from a multitude of youths and young men.

> "'Tis an old maxim in the schools,
> That vanity's the food for fools."

A third motive may be to popularize an institution for the purpose of impressing the community at large that so many young men resort to said college, and so large numbers graduate therein, because of the superior abilities of the various professors who compose its faculty. It may be also added that unusual advantages are afforded its students for clinical instruction, etc., etc., etc. Doubtful expedients are apt to be resorted to in order to secure the attendance of a great crowd of young men at the lectures ; as clap trap circulars addressed to respectable men all over the country with the assumption that such an institution has advantages over all others, and from the large numbers who annually attend the lectures, its faculty can afford to favor worthy young men of limited means by admitting them at greatly reduced rates, or for a nominal fee, to all the privileges and immunities of the college. Possibly by this means hundreds of youngsters irrespective of preliminary education, mental or moral culture, or any other quality pertaining to respectability, are admitted without any question, or upon any conditions except the individual secret that the terms are specially made and fees reduced to suit his convenience, and that this gracious favor is unknown to all the other students and every body else save the two contracting parties.

Let a stranger happen to be in the lecture hall of this miscellaneous and populous medical college during the interim of the lectures, and the absence of all the professors, and he will find himself in a pandemonium,a pell-mell, all kinds of missiles being hurled at one another, amidst a wild and general hurrah of uncouth and uncultured young fellows. What a class of young *gentlemen ?* to choose a medical adviser from !

Is it not a mistaken idea in canvassing promiscuously for a large number of young men to fill a medical college regardless of character, previous education, or aptitude for study ? What must be the outcome from a large and mixed multitude of uncultured medical students at graduation time ? Either one of two things : The consciences of the members of the faculty must be strained to pass many ignorant and incompetent men ; or many of such men, such incompetent students, must endure the sad mortification of being rejected after having spent their time and means in hope of succeeding.

> " There is a kind of conscience some men keep,
> Is like a member that's benumbed with sleep ;
> Which, as it gathers blood, and wakes again,
> It shoots, and pricks, and feels as big as ten."

Would it not be to the credit of any medical faculty in the outset to advise such unpromising young men to decline entering college, or to postpone application for the same until they shall prepare themselves first, for admission, by studying the rudiments of a fair education, and by habits of study for mental and moral culture. It is better for a medical college to graduate fifty competent and high minded good young men, than one hundred and fifty when two-thirds of whom may be unfit to enter upon the practice of physic without any reasonable prospects of success.

Young men applying for admission to a collegiate course of medical lectures, should possess a liberal and fair education, and should produce a certificate of their proficiency in the English branches, natural sciences, and other studies. In the last catalogue of the "Medical Department of the University of Pennsylvania," it is announced that, "A preliminary examination is now required of all candidates who have not received a collegiate degree, or passed the matriculation examination of a recognized college, or who do not present a certificate covering the required subjects from a recognized normal or high school. The candidate for admission will be required: *First*, to write a brief essay not exceeding a page of foolscap, which will serve as a test of his qualifications in orthography and grammar. *Second*, to undergo an examination in the elementary principles of physics, or the subjects considered in Part I, of Towne's Chemistry."

The wisdom of the Pennsylvania University is apparent. Some other medical colleges may have adopted similar requirements. It should be a practice or custom universal in all medical schools. A thorough preliminary education will greatly facilitate the collegiate course of a medical education.

Candidates for a course of medical lectures should possess both mental and moral culture. They should have good minds, be studious in their habits, and particularly should they possess good morals, and be entirely exempt from dissipation of every kind. They should be teachable and gentle.

The course of study in the schools should be prolonged beyond the usual term of five months in two winters each. The total has been ten months of public instruction. Where three courses of lectures have been adopted of five months each, the total number of months of public instruction is fifteen months, during the three winters. If a school should extend the time of each term seven and a half months, say from the middle of Septembet to May the first, following, then two terms would sum up fifteen months, or the same length of time for attending the public lectures as is required by the schools which have adopted three courses of five months each. The Pennsylvania University, already referred to, has announced that two years hence, it will extend its sessions to the 15th of April, which will make six and a half months instead of five months as at present.

This subject will be further discussed in a succeeding article.

